# A Nomogram for Predicting the Risk of Radiotherapy-Related Esophageal Fistula in Esophageal Cancer Patients

**DOI:** 10.3389/fonc.2021.785850

**Published:** 2022-01-18

**Authors:** Zhongxuan Gui, Huiquan Liu, Weijiong Shi, Yuechen Xu, Han Qian, Fan Wang

**Affiliations:** Department of Radiation Oncology, The First Affiliated Hospital of Anhui Medical University, Hefei, China

**Keywords:** esophageal cancer, radiotherapy, esophageal fistula, risk factors, nomogram

## Abstract

**Background:**

To construct and validate a nomogram for predicting the risk of esophageal fistula in esophageal cancer patients receiving radiotherapy.

**Methods:**

A retrospective nested case–control study was performed, in which a total of 81 esophageal fistula patients and 243 controls from 2014 to 2020 in the First Affiliated Hospital of Anhui Medical University were enrolled. Factors included in the nomogram were determined by univariate and multiple logistic regression analysis. The following methods including ROC curve, C-index, calibration curves, Brier score, and decision curve analysis (DCA) were adopted to evaluate this nomogram.

**Results:**

Multivariate logistic regression analysis showed that T4 stage, level 4 stenosis, ulcerative esophageal cancer, prealbumin, and maximum diameters of GTV and NLR were the independent risk factors of esophageal fistula. Accordingly, a nomogram incorporating the aforementioned six parameters was constructed. The AUC was 0.848 (95% CI 0.901–0.895), indicating a high prediction accuracy of this nomogram. Further evaluation of this model showed that the C-index was 0.847, while the bias-corrected C-index after internal validation was 0.833. The Brier score was 0.127. The calibration curves presented good concordance, and the DCA revealed promising clinical application.

**Conclusions:**

The nomogram presents accurate and applicable prediction for the esophageal fistula risk in esophageal cancer patients receiving radiotherapy.

## Introduction

Esophageal cancer (EC) is the seventh most common malignancy worldwide leading to estimated 544,000 deaths in 2020 (1). Patients with EC are usually diagnosed at the advanced or metastatic stage due to the lack of early symptoms and the rapid progression of carcinoma. Thus, a considerable proportion of EC patients are considered inoperable or surgically contraindicated at the initial visit. Radiotherapy, especially the intensity-modulated radiation therapy (IMRT), plays a critical role in the treatment of locally advanced inoperable EC (2). It is remarkable that esophageal fistula (EF), a fatal treatment-related complication, may occur during and after radiotherapy. The incidence of EF in EC patients receiving chemoradiotherapy is about 4.3%–22% according to previous studies (3–10). The common clinical symptoms of EF include bucking, back/chest/abdominal pain, fever, hydrothorax, dysphagia, and empyema (11). Therefore, early prediction of EF and appropriate intervention are important to enhance clinical outcomes and increase quality of life.

Previous literature (6, 8, 12) described that several clinical parameters are closely correlated with the occurrence of EF, including age, T stage, N stage, stenosis, ulceration, low serum cholesterol level, and body mass index (BMI). However, a unified diagnosis criterion for esophageal stenosis has not been unified, and the majority of studies defined stenosis solely based on symptoms (4, 8, 13). To date, there are still no reliable clinical standards for predicting high-risk EF. In this study, we further refined several EF-associated parameters and explored a clinically applicable nomogram to predict EF risk for EC patients receiving radiotherapy.

## Materials and Methods

### Study Design

We retrospectively studied the medical records of EC patients receiving radiotherapy in the First Affiliated Hospital of Anhui Medical University, between October 19, 2014, and June 15, 2020. Follow-up was carried out since the radiotherapy stated until the EF occurred or until June 15, 2021, ensuring that each patient was followed for sufficient time to accurately assess the occurrence of EF. The enrolled EC patients with previous malignancies, history of esophageal surgery, already formed fistula before treatment, and lost follow-up were excluded. The inclusion criteria for EF patients are applied: (1) histologically proved squamous cell carcinoma, adenocarcinoma, or small cell carcinoma of the esophagus; (2) complete record of the necessary clinical characteristics; (3) clinically confirmed EF or esophageal perforation which were detected by endoscopy, computed tomography (CT), or esophagography; and (4) no EF before radiotherapy. The diagnostic standards of EF were as follows: (i) iodine examination shows that contrast media leak out from the patient’s fistula, or into the patient’s chest, mediastinum; (ii) CT scan findings include mediastinal air surrounding the esophagus, abscess cavities adjacent to the esophagus in the pleural space, mediastinal air, pleural effusion, pneumothorax, and subdiaphragmatic air(11). To improve the comparison and the stability of the results, the cases and controls were matched by age, gender, and diagnosis time at a ratio of 1:3. This retrospective nested case–control study was approved by the institutional research ethics committee of Anhui Medical University.

### Data Collection

In this single-centered, retrospective study, we obtained the demographic characteristics, laboratory data, radiological examinations, and therapeutic strategy from electronic medical records. The following clinical characteristics were collected before radiotherapy: general characteristics (gender, age, body mass index (BMI), smoking history, hypertension, diabetes (DM)), tumor characteristics (stage, location, ulcerative EC, esophageal stenosis), treatment characteristics (re-radiotherapy, radiotherapy dose, chemotherapy, gross tumor volume (GTV), maximum diameter of GTV, length of GTV, treatment response), and hematological data (albumin, hemoglobin, prealbumin, neutrophil count, lymphocyte count).

The pretreatment clinical staging was on the basis of the American Joint Committee on Cancer (AJCC) 8th edition staging system (14). GTV was defined by the planning physicians as the primary tumor (GTVp) and involved mediastinal and hilar nodes (GTVn) found by computed tomography (CT) or positron emission tomography/computed tomography (PET/CT) before treatment. The NLR was defined as the absolute neutrophil count divided by the absolute lymphocyte count. The treatment response was assessed 30 days after radiotherapy by enhanced CT based on Response Evaluation Criteria in Solid Tumors (RECIST) version 1.1, and it was classified as clinically complete response (CR), partial response (PR), stable disease (SD), or progressive disease (PD). CR was defined as the disappearance of all target lesions, PR as reduction by 30% or more in maximum diameter of target lesion, PD as increase by 20% or more in the longest tumor diameter of target lesion or appearance of new lesions, and SD as other than CR, PD, and PR.

**Figure 1:**
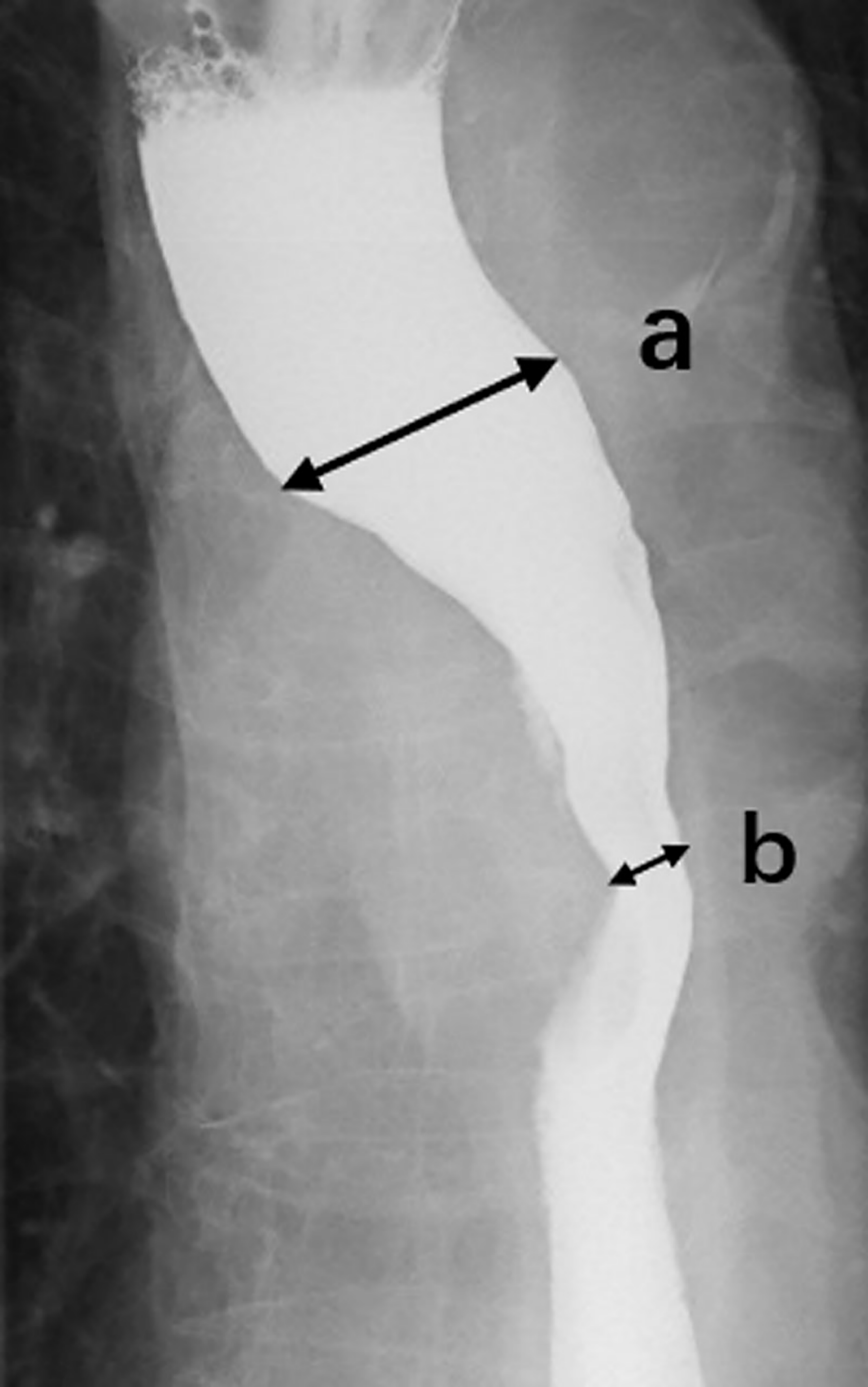
Esophagography image. We reviewed the esophagography image obtained before radiotherapy and measured the lumen diameter at the widest part **(A)** of the oral side and the narrowest part **(B)** of the lesion, then calculated the stenotic ratio (c = (a - b)/a * 100).

To determine the stenosis of esophagus, we reviewed the esophagography image obtained before radiotherapy and measured the lumen diameter at the widest part of the oral side (**Figure 1A**) and the narrowest part of the lesion (**Figure 1B**). The stenosis ratio was calculated as following formula: c = (a - b)/a * 100%. The severity of esophageal stenosis (stenosis ratio) was evaluated and classified as the following grades: grade I, 0%–24%; grade II, 25%–49%; grade III, 50%–74%; grade IV, 75%–100%.

### Statistical Analyses

Odds ratios (OR) with 95% confidence intervals (CI) were used to evaluate the relationship between the clinical parameters and EF, and the best cutoff values to predict EF risk were determined using receiver operating characteristic (ROC) curves. We investigated all the clinical factors by univariable logistic regression for paired samples, and the significant factors were included in the multivariable logistic-regression model. Statistical analyses were performed using IBM SPSS 22.0 software. A nomogram integrating independent risk factors of EF was created using R software (version 3.6.1). The receiver operating characteristic (ROC) curve and the area under the ROC curve (AUC) were applied to assess the discrimination of the model. We adopted three methods, including C-index for discrimination, calibration curves, and Brier score to evaluate this nomogram. The established nomogram was further internally validated by bootstrapping (1,000 bootstrap replicates) to obtain bias-corrected predictive parameters. Significance was defined as 2-sided *p-*value of < 0.05.

## Results

### Characteristics of Participants

Between October 19, 2014, and June 15, 2020, 1,894 cases who had undergone radiotherapy were identified in our database and 711 cases were excluded based on our exclusion criteria. Among the 1,183 EC patients, 81 (6.85%) had developed EF before June 15, 2021. After matching by age, gender, and diagnosis time, a total of 324 EC patients, including those 81 (25.0%) EF cases and 243 (75.0%) controls, were enrolled for the subsequent analyses. The follow-up period ranged from 2.3 to 82.6 months, and the median time was 51.7 months. The median age of these participants was 70.0 years, and the male-to-female ratio is 3.3:1. Middle thoracic (40.7%) EC was more common than upper thoracic (31.1%) and lower thoracic (28.0%) EC. Of these patients who developed EF, 12 patients suffered perforation during RT, while 69 patients developed this complication after RT. The median intervals between the end of radiotherapy and the EF onset were 4.60 months (95% CI: 3.50–5.64). Among all the 81 cases with EF, 46 cases developed esophagomediastinal fistula, 28 cases developed esophagotracheal fistula, 2 cases developed esophago-arterio fistulas, and 5 cases suffered both esophagomediastinal and esophagotracheal fistula. Managements of fistula included nutrient canal in 61 patients (75.3%), esophageal stent in 16 patients (19.7%), and parenteral nutrition in 4 patients (4.9%). The patient characteristics are listed in **Table 1**.

**Table 1:** The characteristics of patients with radiotherapy-related esophageal fistula.

Characteristics	No esophageal fistula	Esophageal fistula
Gender		
Male	186 (0.77)	62 (0.77)
Female	57 (0.23)	19 (0.23)
Age (years)		
<60	41 (0.17)	20 (0.25)
≥60	202 (0.83)	61 (0.75)
History of smoking		
No	168 (0.69)	48 (0.59)
Yes	75 (0.31)	33 (0.41)
History of hypertension		
No	212 (0.87)	67 (0.83)
Yes	31 (0.13)	14 (0.17)
History of diabetes		
No	235 (0.97)	76 (0.94)
Yes	8 (0.03)	5 (0.06)
BMI (kg/m^2^)		
<20	93 (0.38)	43 (0.53)
≥20	150 (0.62)	38 (0.47)
T stage		
T1–3	224 (0.92)	55 (0.68)
T4	19 (0.08)	26 (0.32)
N stage		
N0	104 (0.43)	25 (0.31)
N1–3	139 (0.57)	56 (0.69)
M stage		
M0	189 (0.78)	66 (0.81)
M1	54 (0.22)	15 (0.19)
Location of primary tumor		
Upper thoracic esophagus	74 (0.30)	27 (0.33)
Middle thoracic esophagus	95 (0.39)	37 (0.46)
Lower thoracic esophagus	74 (0.30)	17 (0.21)
Ulcerative tumor		
No	191 (0.79)	34 (0.42)
Yes	52 (0.21)	47 (0.58)
Maximum diameter of GTV (cm)		
≤2.5	72 (0.30)	7 (0.09)
>2.5	171 (0.70)	74 (0.91)
Length of GTV (cm)		
≤5.5	141 (0.58)	28 (0.35)
>5.5	102 (0.42)	53 (0.65)
GTV volume (cm^3^)		
≤60	151 (0.62)	36 (0.44)
>60	92 (0.38)	45 (0.56)
Fraciton dose (Gy)		
1.8	17 (0.07)	10 (0.12)
2.0	226 (0.93)	71 (0.88)
Total radiation dose		
<60	148 (0.58)	36 (0.54)
≥60	92 (0.42)	45 (0.46)
Re-radiotherapy		
No	231 (0.95)	70 (0.86)
Yes	12 (0.05)	11 (0.14)
Treatment modalities		
Concurrent CRT	127 (0.52)	38 (0,47)
Sequential CRT	103 (0.42)	39 (0.48)
Without CT	13 (0.05)	4 (0.05)
Treatment response		
SD+PD	106 (0.44)	31 (0.38)
CR+PR	137 (0.56)	50 (0.62)
Stenosis before radiotherapy		
Levels 1–3	151 (0.62)	18 (0.22)
Level 4	92 (0.38)	63 (0.78)
Hemoglobin (g/L)		
<120	89 (0.37)	42 (0.52)
≥120	154 (0.63)	39 (0.48)
Albumin (g/L)		
<35	50 (0.21)	23 (0.28)
≥35	193 (0.79)	58 (0.72)
Prealbumin (mg/L)		
<180	66 (0.27)	37 (0.46)
≥180	177 (0.73)	44 (0.54)
NLR		
<3.2	130 (0.53)	30 (0.37)
≥3.2	113 (0.47)	51 (0.63)
PLR		
<155	135 (0.56)	27 (0.33)
≥155	108 (0.44)	54 (0.67)

BMI, body mass index; GTV, gross tumor volume; CRT, chemoradiotherapy; CT, chemotherapy; SD, stable disease; PD, progressive disease; CR, complete response; PR, partial response; NLR, neutrophil to lymphocyte ratio; PLR, platelet to lymphocyte ratio.

### Risk Factors for EF

As shown in **Table 2**, univariate analysis revealed that BMI < 20 kg/m^2^, N1–3 stage, T4 stage, NLR, hemoglobin, prealbumin, re-radiotherapy, ulcerative EC, stenosis, length of GTV, and maximum diameter of GTV were significantly correlated with the occurrence of EF (*p*-value < 0.05). The other clinical parameters including age, albumin, tumor location, M stage, total dose > 60 Gy, single dose, GTV volume, chemotherapy, treatment response (PR+CR vs. SD+PD), smoking history, diabetes, and hypertension were not significant for their association with EF. Multivariate analysis showed that T4 stage, level 4 stenosis, ulcerative EC, prealbumin, and maximum diameters of GTV and NLR remained significant (*p-*value < 0.05), which indicated that these clinical characteristics were independent risk factors for the occurrence of EF (**Table 2**).

**Table 2:** Univariate and multivariate analysis of the factors associated with esophageal fistula.

Characteristics	Univariate			Multivariate		
	OR	95% CI	*p*-value	OR	95% CI	*p*-value
Age (years)						
<60	0.621	0.338–1.143	0.126			
≥60						
History of smoking						
No	0.990	0.565–1.737	0.973			
Yes						
History of hypertension						
No	0.574	0.249–1.324	0.193			
Yes						
History of diabetes						
No	2.000	0.626–6.393	0.242			
Yes						
BMI (kg/m^2^)						
<20	0.560	0.339–0.924	0.924	0.872	0.404–1.882	0.728
≥20						
T stage						
T1–3	5.278	2.687–10.366	<0.001	5.357	2.052–13.983	0.001
T4						
N stage						
N0	1.787	1.001–3.188	0.049	1.160	0.5–2.691	0.730
N1–3						
M stage						
M0	0.801	0.427–1.504	0.491			
M1						
Location of primary tumor						
Upper thoracic esophagus	1.000		1.000			
Middle thoracic esophagus	1.050	0.590–1.866	0.869			
Lower thoracic esophagus	0.591	0.288–1.214	0.152			
Ulcerative tumor						
No	5.504	3.015–10.049	<0.001	3.102	1.536–6.265	0.002
Yes						
Maximum diameters of GTV (cm)						
≤2.5	4.611	1.999–10.633	<0.001	3.675	1.432–9.433	0.007
>2.5						
Length of GTV (cm)						
≤5.5	2.553	1.510–4.318	<0.001	1.297	0.623–2.698	0.487
>5.5						
GTV volume (cm^3^)						
≤60	2.048	1.226–3.421	0.008	1.378	0.678–2.8	0.375
>60						
Fraction dose (Gy)						
1.8	0.492	0.202–1.198	0.118			
2.0						
Total radiation dose						
<60	1.149	0.695–1.899	0.589			
≥60						
Re–radiotherapy						
No	2.887	1.244–6.702	0.014	2.599	0.707–9.548	0.150
Yes						
Treatment modalities						
Concurrent CRT	1.000					
Sequential CRT	1.038	0.319–3.373	0.951			
Without CT	1.258	0.737–2.146	0.400			
Treatment response						
SD+PD	1.217	0.732–2.023	0.449			
CR+PR						
Stenosis before radiotherapy						
Level 1–3	5.631	3.069–10.331	<0.001	6.549	2.984–14.373	<0.001
Level 4						
Hemoglobin (g/dL)						
<120	0.528	0.311–0.898	0.018	0.834	0.4–1.738	0.627
≥120						
Albumin(g/dL)						
<35	0.678	0.388–1.185	0.173			
≥35						
Prealbumin (mg/L)						
<180	0.439	0.257–0.749	0.003	0.399	0.189–0.842	0.016
≥180						
NLR						
<3.2	1.953	1.158–3.293	0.012	2.326	1.12–4.831	0.024
≥3.2						
PLR						
<155	2.657	1.516–4.659	0.001	1.492	0.609–3.657	0.382
≥155						

BMI, body mass index; GTV, gross tumor volume; CRT, chemoradiotherapy; CT, chemotherapy; SD, stable disease; PD, progressive disease; CR, complete response; PR, partial response; NLR, neutrophil to lymphocyte ratio; PLR, platelet to lymphocyte ratio.

### Predictive Nomogram for EF

**Figure 2:**
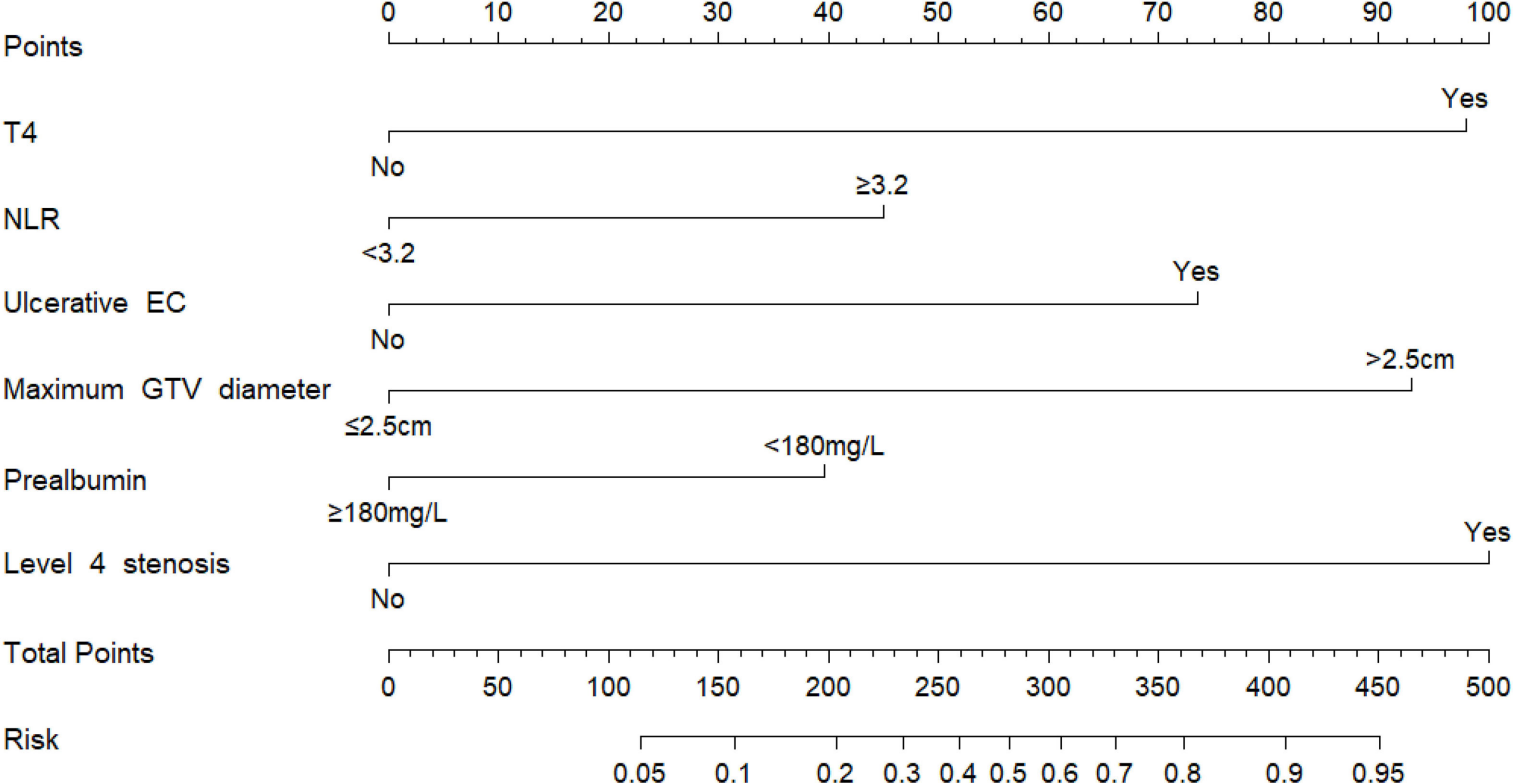
Nomogram for the individualized prediction of radiation-related esophageal fistula in esophageal cancer patients. The nomogram was developed in the cohort, using T4, level 4 stenosis, ulcerative EC, prealbumin, and maximum diameters of GTV and NLR. GTV, gross tumor volume; NLR, neutrophil-to-lymphocyte ratio.

According to the results of multivariate analysis, a nomogram incorporating the 6 independent risk factors was constructed to predict EF (**Figure 2**). The total point was calculated with the use of T4, NLR, ulcerative EC, level 4 stenosis, prealbumin, and maximum diameter of GTV. The point of each of these variables was given a score on the point scale axis. A total score could be easily calculated by adding each single score, and by projecting the total score to the lower total point scale, we were able to estimate the probability of EF.

### Evaluation and Validation the Nomogram

**Figure 3:**
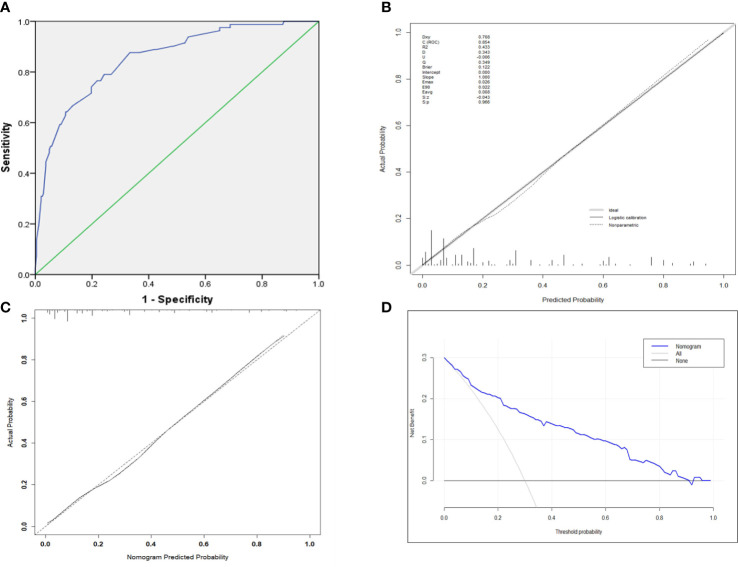
**(A)** ROC curve for the prediction nomogram. **(B)** The Brier score for the prediction nomogram. **(C)** Calibration curve showing nomogram-predicted EF probabilities compared with the actual EF. **(D)** The decision curve analysis of the nomogram.

The AUC was 0.848 (95% CI 0.901–0.895) (**Figure 3A**), indicating robust discrimination. The Brier score of the nomogram was 0.127, which was close to 0, indicating great predictive ability (**Figure 3B**). As shown in **Figure 3C**, the calibration plot showed good conformity between predicted and actual probability for EF. The uncorrected concordance index (C-index) was 0.847, and the corrected C-index generated by internal validation was 0.833 (**Figure 3C**). Finally, we performed a decision curve analysis (DCA) to evaluate the clinical utility of the nomogram and its effective threshold ranged from approximately 7% to 91%, showing that using this nomogram was more effective than the “treat-all” or the “treat-none” strategy in predicting EF when the prediction probability was within this range (**Figure 3D**).

## Discussion

The EF, a severe complication, deteriorates the quality of life and shortens survival in EC patients. Predicting EF risk is crucial for developing individual therapeutic strategies. In this study, we comprehensively evaluated the fistula-related parameters and identified several new independent risk factors. For dosimetry-related indicators, we found that the incidence of fistula was 28.9% in the group of GTV maximum diameters ≤2.5 cm, while the incidence decreased to 14.6% in the group of maximum diameter of GTV > 2.5 cm. To some extent, the maximum diameter of GTV indicated the severity of local radiation damage and the depth of tumor invasion. These results suggest that for the high-risk EF patients, moderate shrinks of GTV are needed.

The status when esophageal carcinoma invades adjacent structures, such as the pleura, pericardium, azygos vein, diaphragm, peritoneum, aorta, vertebral body, and airway, is defined as T4 stage in the 8th Edition of the AJCC TNM Staging System (15). Recently, Chen et al. (16)revealed that the incidence rate of EF was 30.1% in EC patients (stage T4b) and the median overall survival was only 6.9 months. It is easy to understand that if the space-occupying lesions were eliminated speedily by chemoradiotherapy without sufficient tissue repair, the fistula might form between the esophageal lumen and contiguous structures. Formation of a fistula between the esophagus and the mediastinum was suspected.

We believe that EC with external esophageal invasion should receive individualized radiotherapy not only to kill the tumor cells but also to maximize normal tissue repair. Additionally, we also characterized ulcerative lesion as an independent factor of EF, which was consistent with previous studies (8, 16). In our study, we observed that the incidence rate of ulcerative-type carcinoma diagnosed before radiotherapy in the EF group was nearly three times that in the control group (58.0% vs. 21.3%). Ulcerative esophageal cancer has a deep invasion and thin wall, reaching or penetrating the muscular layer, and then the perforation may occur due to increased luminal pressure during swallowing or severe coughing.

It is estimated that more than half of EC patients suffer from malnutrition (17). Cancer-associated dysphagia and anorexia are the leading causes of malnutrition, while radiation-induced mucositis makes matters worse. Malnutrition and cachexia restrain the damage repair, reduce therapeutic effects, and increase mortality (17, 18). In this study, we also explored the risk factors from the perspective of nutrition and found that BMI, hemoglobin, and prealbumin were significantly associated with the occurrence of EF. Meanwhile, multivariable analysis demonstrated that low prealbumin was an independent risk factor, which was not previously reported. Previous studies have shown that prealbumin is considered to be more sensitive than albumin in the nutritional assessment of patients undergoing radiotherapy (19). Serum prealbumin with a half-life of 2 to 3 days in the human body is a good clinical marker of protein balance and nutritional status (20, 21). These results indicate that nutritional support, such as oral nutritional supplements, promisingly prevents the occurrence of EF.

Most of the published articles investigated esophageal stenosis based on symptoms and did not define its degree of severity. The NCCN guidelines noted that the most common cause of dysphagia is obstruction, but it may also be associated with cancer-related dysmotility (22), which may affect the assessment of esophageal stenosis. Thus, we used a specific criterion to evaluate and grade the stenosis by esophageal barium meal examination before treatment. Intriguingly, our results showed that esophageal stenosis at level 4 was a significant independent risk factor in fistula formation. It is speculated that the internal pressure was associated with severity of the esophageal stenosis and caused expansionary damage to esophageal wall. As a result, it is appropriate to identify the esophageal stenosis before radiotherapy, so as to make dietary adjustments and palliative management, such as endoscopic stenting and endoscopic dilation (23, 24).

Malignant tumors usually trigger an intrinsic inflammatory response to establish a tumorigenic microenvironment (25, 26). The NLR as a marker of systemic inflammatory response has received great attention because of its accessibility. In clinical practice, the NLR is increasingly used to predict bacteremia, peptic ulcer perforation, severe cholecystitis, acute cholecystitis, acute pancreatitis, acute coronary syndrome and community-acquired infections, and even the survival of cancer patients (27–30). Systemic inflammatory responses have been proved to influence the motility, invasiveness, and survival of malignant cells through upregulating cytokines, such as IL-1β, IL-6, IL-7, IL-8, and IL-12. The host-cellular response to IL-8 released by cancer cells enhances neutrophil infiltration, which promotes remodeling of the extracellular matrix and tumor progression (31). High NLR represents more severe inflammation and more advanced disease with aggressive clinical characteristics. In this study, we preliminarily explored the significant association of high NLR with EF. Further research is needed to explore the specific mechanism and the application of NLR in EF.

The nomogram is a kind of visual graph based on the multiple regression model. It integrates several parameters and consists of different length line segments. In this study, we screened out six independent risk factors by multiple regression to establish a personalized prediction model. Further validation proved that this nomogram has good predictive accuracy and clinical application potential. This is the first and comprehensive calculable tool consisting of systemic inflammatory status, nutritional status, and radiation-related parameters to predict EF risk. However, our current study has certain drawbacks that merit discussion. First, as a retrospective, single-center study, it was inevitable to have potential bias. Second, only internal validation was carried out due to limited EF cases. External validation from other centers is necessary to confirm the clinical value of this nomogram. Lastly, the interaction between inflammation and fistula remains obscure, and more trials are needed to clarify the underlying mechanisms. In view of these limitations, we are now planning to expand the sample size of EF patients, further explore predictors with clinical practicability, and improve the model on the basis of the current findings to optimize the prediction of EF.

In summary, we characterized several new clinical parameters as the independent risk factors of EF. A nomogram was accordingly constructed and visualized to facilitate the prediction of EF risk. This calculable tool is promisingly applied in clinical practice to participate in determining individual therapeutic strategies for EC patients.

## Data Availability Statement

The original contributions presented in the study are included in the article/supplementary material. Further inquiries can be directed to the corresponding author.

## Ethics Statement

The studies involving human participants were reviewed and approved by the Institutional Review Board of the First Affiliated Hospital of Anhui Medical University. Written informed consent for participation was not required for this study in accordance with the national legislation and the institutional requirements.

## Author Contributions

ZG and HL conceived and designed this study. WS, YX, and HQ processed the data analysis. ZG wrote the article. FW revised the final manuscript. All authors contributed to the article and approved the submitted version.

## Conflict of Interest

The authors declare that the research was conducted in the absence of any commercial or financial relationships that could be construed as a potential conflict of interest.

## Publisher’s Note

All claims expressed in this article are solely those of the authors and do not necessarily represent those of their affiliated organizations, or those of the publisher, the editors and the reviewers. Any product that may be evaluated in this article, or claim that may be made by its manufacturer, is not guaranteed or endorsed by the publisher.
